# Supplementary material for: Deletion of wfs1 Impairs Oligodendrocyte Precursor Cells Dorsal Distribution and Myelination Through the wfs1-hmgcs1 Axis in Zebrafish
Source: Biology (Basel). 2026 Aug 16;15(16):1402. doi: 10.3390/biology15161402 (PMC13510151; doi:10.3390/biology15161402)
Supplement: Supplementary file 1 [file biology-15-01402-s001.zip › File S1-Transcriptome sequencing data and differentially expressed genes (DEGs) in wfs1-- zebrafish at 5 dpf/2.KEGG/wfs1KOvsWT/GSEA/WT/SELENOCOMPOUND_METABOLISM/SELENOCOMPOUND_METABOLISM(DRE00450).html]

Details for gene set SELENOCOMPOUND\_METABOLISM(DRE00450)[GSEA]

|  || Dataset | deg.sample |
| Phenotype | sample.cls |
| Upregulated in class | WT |
| GeneSet | SELENOCOMPOUND\_METABOLISM(DRE00450) |
| Enrichment Score (ES) | -0.39918196 |
| Normalized Enrichment Score (NES) | -1.4909955 |
| Nominal p-value | 0.034068137 |
| FDR q-value | 0.60131055 |
| FWER p-Value | 0.722 |
Table: GSEA Results Summary

  

Fig 1: Enrichment plot: SELENOCOMPOUND\_METABOLISM(DRE00450)      
 Profile of the Running ES Score & Positions of GeneSet Members on the Rank Ordered List

  

| PROBE | DESCRIPTION (from dataset) | GENE SYMBOL | GENE\_TITLE | RANK IN GENE LIST | RANK METRIC SCORE | RUNNING ES | CORE ENRICHMENT || 1 | 103911233 | LOC103911233 |  |  | 562 | 2.827 | 0.0889 | No |
| 2 | 101884223 | LOC101884223 |  |  | 682 | 2.621 | 0.1839 | No |
| 3 | 445818 | cthl |  |  | 799 | 2.387 | 0.2702 | No |
| 4 | 103911241 | LOC103911241 |  |  | 1022 | 2.074 | 0.3413 | No |
| 5 | 324947 | sephs1 |  |  | 3798 | 0.868 | 0.2870 | No |
| 6 | 378847 | mtr |  |  | 9320 | 0.346 | 0.1267 | No |
| 7 | 80939 | papss2b |  |  | 11321 | 0.226 | 0.0725 | No |
| 8 | 352933 | sps2 |  |  | 14627 | 0.064 | -0.0288 | No |
| 9 | 791592 | ccbl1 |  |  | 15939 | 0.001 | -0.0699 | No |
| 10 | 393123 | sepsecs |  |  | 17049 | -0.034 | -0.1034 | No |
| 11 | 352924 | txnrd1 |  |  | 17188 | -0.040 | -0.1062 | No |
| 12 | 566565\_2 | mars2 |  |  | 22951 | -0.324 | -0.2748 | No |
| 13 | 338183 | mars |  |  | 22995 | -0.327 | -0.2639 | No |
| 14 | 322055 | cth |  |  | 23997 | -0.391 | -0.2805 | No |
| 15 | 559537 | scly |  |  | 26112 | -0.538 | -0.3266 | No |
| 16 | 798259 | si:ch1073-179p4.3 |  |  | 26528 | -0.581 | -0.3177 | No |
| 17 | 559488 | papss1 |  |  | 28123 | -0.759 | -0.3391 | No |
| 18 | 777719\_1 | papss2a |  |  | 30038 | -1.262 | -0.3517 | Yes |
| 19 | 393315 | ccbl2 |  |  | 30227 | -1.353 | -0.3066 | Yes |
| 20 | 103911967 | LOC103911967 |  |  | 30478 | -1.493 | -0.2582 | Yes |
| 21 | 777719 | papss2a |  |  | 31559 | -3.081 | -0.1760 | Yes |
| 22 | 571201 | si:ch73-97h19.2 |  |  | 31873 | -4.947 | 0.0005 | Yes |
Table: GSEA details [plain text format]

  

Fig 2: SELENOCOMPOUND\_METABOLISM(DRE00450)      
 Blue-Pink O' Gram in the Space of the Analyzed GeneSet

  

Fig 3: SELENOCOMPOUND\_METABOLISM(DRE00450): Random ES distribution      
 Gene set null distribution of ES for **SELENOCOMPOUND\_METABOLISM(DRE00450)**
